# Pharmacological management of late-onset major depression – Evidence-based approaches from the first episode to treatment-resistant cases

**DOI:** 10.1192/j.eurpsy.2025.1379

**Published:** 2025-08-26

**Authors:** O. Vasiliu, A. M. Ciobanu, B. M. Petrescu, A. G. Mangalagiu

**Affiliations:** 1 Neuroscience Department, “Carol Davila” University of Medicine; 2 Psychiatry Department, “Dr. Carol Davila” University Emergency Central Military Hospital; 3 Psychiatry Department, “Prof. Dr. Alexandru Obregia” Clinical Hospital of Psychiatry, Bucharest, Romania

## Abstract

**Introduction:**

Depression in older adults may present itself as a recurring disorder originating from earlier life, as a new onset depression (late-onset depression, LOD), or as a depression due to organic diseases (e.g., vascular depression). An estimated 30% of all cases of depression in older adults are represented by LOD. Low tolerability due to the changes of the pharmacodynamic and pharmacokinetic profile (e.g., lower volume of distribution, reduction of renal clearence, longer half-time, different receptoral sensitivity and density), as well as low efficacy of antidepressants and high risk of completed suicide, have been reported in this population.

**Objectives:**

To explore the current state of evidence for the pharmacological treatment of LOD through a narrative literature review.

**Methods:**

This review included three databases (Web of Science/Clarivate, PubMed, and Cochrane), explored from their inception to June 2024, for papers published in English using the keywords “late-onset depression,” “geriatric depression,” “old age depression,” and “antidepressants” or “treatment.”

**Results:**

Based on the results of 27 selected primary and secondary reports, selective serotonin reuptake inhibitors (SSRIs), especially escitalopram and sertraline, are considered the first line of treatment for LOD, with >50% rate of responsiveness being reported. SSRIs are followed by serotonin-norepinephrine reuptake inhibitors (SNRIs), and other new-generation antidepressants, mainly mirtazapine, vortioxetine, and bupropion. The therapeutic guidelines recommend the correct treatment of comorbid neurocognitive disorders; however, the available evidence shows that antidepressants have very limited efficacy in the presence of dementia. The number needed to treat (NNT) for LOD was between 6.7 and 14.4 (Alexopoulos *Transl Psychiatry* 2019;188 9). For treatment-refractory LOD (TRLOD), augmentation with lithium, aripiprazole, memantine, or methylphenidate, as well as switching to phenelzine, nortriptyline, desipramine, or a combination of antidepressants, such as bupropion and nortriptyline have been suggested as potential solutions, but the evidence to support such recommendations has low quality. Regarding the tolerability of antidepressants in this specific population, electrolyte imbalance, worsening of cognitive dysfunction, increased coagulation time, and falls should be considered.

**Conclusions:**

SSRIs are the first-line pharmacological approach in LOD, with SNRIs and other new-generation antidepressants as the second-line. The pharmacological recommendations for TRLOD are based on low-quality data. Due to the tolerability and safety-specific aspects, special care should be given when monitoring these patients during the pharmacological treatment.

**Disclosure of Interest:**

None Declared

